# Altered Cerebellar Spontaneous Activity and Its Association with Arousal Index in Comorbid Insomnia and Obstructive Sleep Apnea: A Resting-State fMRI Study

**DOI:** 10.3390/jcm15083080

**Published:** 2026-04-17

**Authors:** Jiaming Huang, Qianqian Gao, Yanting Zhang, Rui Song, Sheng Shi, Xiaochuan Cui, Xiangming Fang, Yunyun Zhang

**Affiliations:** 1 Department of General Practice and Sleep Center, The Affiliated Wuxi People’s Hospital of Nanjing Medical University, Wuxi People’s Hospital, Wuxi Medical Center, Nanjing Medical University, Wuxi 214000, China; hjmnmu@163.com (J.H.); crayon_z06@163.com (Y.Z.); 18774860252@163.com (R.S.); m19907432473@163.com (S.S.); 2 Department of Medical Imaging, The Affiliated Wuxi People’s Hospital of Nanjing Medical University, Wuxi People’s Hospital, Wuxi Medical Center, Nanjing Medical University, Wuxi 214000, China; gaoqian123011@163.com (Q.G.); xiangming_fang@njmu.edu.cn (X.F.)

**Keywords:** insomnia, obstructive sleep apnea, COMISA, magnetic resonance imaging, neuroimaging

## Abstract

**Background**: Frequent nocturnal arousals are a core feature of comorbid insomnia and obstructive sleep apnea (COMISA), yet the underlying central mechanisms remain unclear. Identifying brain functional correlates of nocturnal awakenings may help clarify arousal-related mechanisms and inform potential interventional targets. **Methods**: A total of 99 participants (COMISA, insomnia alone, OSA alone, and healthy controls) underwent clinical assessments, polysomnography, and brain magnetic resonance imaging (MRI). MRI metrics were compared across groups, followed by correlation and regression analyses with the arousal index, adjusting for respiratory events and insomnia-related factors. **Results**: Patients with COMISA exhibited more severe insomnia symptoms, greater daytime dysfunction, and more frequent nocturnal awakenings than those with insomnia alone, although their arousal index did not differ from that of the OSA group. Patients with COMISA exhibited altered activity in the right cerebellar lobule VIII (Cerebelum_8_R), left middle temporal gyrus, and right inferior frontal gyrus, opercular part. Lower fractional amplitude of low-frequency fluctuations (fALFF) in the Cerebelum_8_R was associated with a higher arousal index. This association remained significant after controlling for insomnia severity and sleep efficiency but was attenuated after adjustment for AHI. **Conclusions**: Reduced functional activity in the Cerebelum_8_R was independently associated with sleep fragmentation in COMISA, independent of insomnia severity but potentially mediated by respiratory events. These findings suggest this region may be involved in arousal-related neural regulation and could represent a therapeutic target for the complex symptoms of COMISA. Trial Registration: Chinese Clinical Trial Registry, ChiCTR2500095809.

## 1. Introduction

Two prevalent sleep disorders, insomnia and obstructive sleep apnea (OSA), often co-occur and lead to increased disease severity [1]. Compared with either disorder alone, comorbid insomnia and sleep apnea (COMISA) is associated with more severe sleep disturbance, daytime dysfunction, mood disorder, and cognitive impairment [2,3,4,5], as well as significantly increased risks of hypertension, cardiovascular disease, and all-cause mortality [6,7,8]. In children, COMISA occurs in approximately 13–16% of sleep clinic referrals and is also associated with increased anxiety and daytime sleepiness [9,10]. Despite its high prevalence and significant clinical impact in neuropsychiatry, the management of COMISA remains a challenge. Cognitive behavioral therapy for insomnia (CBT-I) and continuous positive airway pressure (CPAP) are commonly used treatment modalities. However, frequent nocturnal awakenings can reduce patients’ willingness to use CPAP, and the discomfort while using the device further lowers the adherence to CPAP and CBT-I [11,12,13]. Hyperarousal is a key mechanism underlying both insomnia and poor sleep quality, in which pre-sleep cognitive arousal plays a significant role in sustaining and activating the insomnia–hyperarousal network [14]. Sarzetto et al. further demonstrated that sleep–wake disruption is associated with the onset of mood disorders [15].

Understanding the mechanisms underlying frequent nocturnal awakenings in patients with COMISA is particularly important. Studies have reported that the respiratory arousal threshold is lower in patients with COMISA than in those with OSA alone, which may partly explain the increased frequency of nocturnal arousals in COMISA [16]. However, a larger cohort study showed no significant difference in arousal threshold between COMISA and OSA groups, indicating that frequent nocturnal arousals in COMISA cannot be fully explained by a reduced threshold [17]. Brooker et al. observed that cortical activity during respiratory events was higher in patients with COMISA than in those with OSA. This increase in cortical activation appears to reflect a state of hyperarousal, resembling patterns commonly described in insomnia [16]. Both conditional arousal and respiratory-driven arousal are considered important contributors to sleep disruption in patients with COMISA. However, it remains unclear how these objective arousal events are linked to alterations in brain functional activity.

Previous studies have indicated that chronic insomnia is associated with hyperactivation of the default mode network (DMN), functional hyperactivity in limbic structures, such as the amygdala, and impaired inhibitory control of the prefrontal cortex [18]. In contrast, OSA is related to hippocampal atrophy due to intermittent hypoxia, disrupted brain network connectivity, and reduced white matter integrity [19,20,21]. Resting-state functional magnetic resonance imaging (rs-fMRI) can detect abnormalities in spontaneous neural activity and functional coordination using blood oxygen level-dependent (BOLD) signal metrics [22,23]. Research on brain activity in patients with COMISA remains scarce, particularly regarding the neural mechanism association with frequent nocturnal awakenings. This study examined the arousal index and rs-fMRI findings in patients with COMISA to illustrate the association between nocturnal awakening and brain functional activity. These findings may offer initial insights into brain regions involved in neuroimaging-based regulation and could help guide future decisions regarding targeted treatments.

## 2. Materials and Methods

### 2.1. Participants

This study was approved by the Institutional Review Board of the Affiliated Wuxi People’s Hospital of Nanjing Medical University (No. KY24152) on 1 March 2024. The registration application for the study protocol was submitted to the Chinese Clinical Trial Registry after ethical approval. Participant recruitment proceeded concurrently with the registration review process, and participants were enrolled consecutively from March 2024 to February 2025. The final registration date was 13 January 2025 (registration number: ChiCTR2500095809). This is an observational study with no intervention. All procedures were conducted in accordance with the Declaration of Helsinki, and written informed consent was obtained from all participants.

The inclusion criteria were as follows: (1) age 18–70 years, right-handedness, and no gender restriction; (2) a diagnosis of OSA according to the International Classification of Sleep Disorders, Third Edition (ICSD-3), confirmed by polysomnography (PSG) with an apnea–hypopnea index (AHI) ≥ 5 events/h and accompanied by typical nocturnal and daytime symptoms [24]; (3) chronic insomnia diagnosed per ICSD-3 criteria, defined as difficulty initiating sleep, maintaining sleep, or early morning awakening, with symptoms persisting for at least three months at a frequency of at least three nights per week, despite adequate opportunity for sleep, accompanied by significant daytime impairment or distress [24]; and (4) absence of psychiatric disorders, history of potent sedative (including opioids and anesthetics) use, or substance/alcohol dependence. Patients with COMISA were required to meet all four criteria; those with insomnia alone had to meet criteria (1), (3), and (4); those with OSA alone had to meet criteria (1), (2), and (4); and healthy controls were required to meet criteria (1) and (4) and to report normal sleep without any comorbidities affecting sleep. The exclusion criteria were as follows: (1) history of severe medical conditions such as malignancy or stroke; (2) presence of other sleep disorders, including sleep paralysis or narcolepsy; and (3) inability to undergo a brain magnetic resonance imaging (MRI).

Clinical and demographic data were collected on the night of polysomnography. Age, sex, body mass index (BMI), neck circumference, waist circumference, and blood pressure were measured by trained staff using standardized equipment. Years of education, medication use, smoking and alcohol consumption, and medical history (hypertension, diabetes, hyperlipidemia, and family history of OSA) were obtained through structured questionnaires administered by the same personnel.

### 2.2. Subjective Assessments of Sleep, Daytime Function, Mood, and Cognition

After the collection of general information, all participants completed a comprehensive set of assessments to evaluate sleep quality, daytime functioning, handedness, mood, and cognitive status. Sleep quality was assessed using the Pittsburgh Sleep Quality Index (PSQI; total score 0–21, with higher scores indicating worse sleep quality; >5 indicates poor sleep quality), the Athens Insomnia Scale (AIS; total score 0–24; ≥6 suggests clinical insomnia), and the Insomnia Severity Index (ISI; total score 0–28; scores 0–7 indicate no clinically significant insomnia, 8–14 subthreshold, 15–21 moderate clinical insomnia, and 22–28 severe clinical insomnia). OSA risk and daytime sleepiness were evaluated using the STOP-Bang Questionnaire (eight yes/no items; one point per “yes”; total score ≥ 3 or ≥2 on the first four items indicates high OSA risk) and the Epworth Sleepiness Scale (ESS; total score 0–24; >6 indicates sleepiness, >11 excessive sleepiness, and >16 dangerous sleepiness), respectively. Fatigue severity was measured with the Fatigue Severity Scale (FSS; total score 9–63; ≥36 indicates significant fatigue). Handedness was confirmed using the Edinburgh Handed-ness Inventory (EHI). Mood status was screened using the Hamilton Anxiety Rating Scale (HAMA; ≥7 suggesting possible anxiety, and ≥14 indicating clinically significant anxiety) and the Hamilton Depression Rating Scale (HAMD; ≥7 suggesting possible depression, and ≥17 indicating clinically significant depression). Cognitive function was assessed with the Mini-Mental State Examination (MMSE) and Montreal Cognitive Assessment-Basic (MoCA-B). For the MMSE, cognitive impairment was defined using education-adjusted cut-off scores: ≤17 for illiterate individuals or those with primary school education, ≤20 for those with middle school education, and ≤24 for those with college education or above. For the MoCA, one point was added for participants with fewer than 12 years of education, and a score of <26 was considered indicative of cognitive impairment. Participants scoring below the cut-off values on either instrument were considered to have cognitive impairment on screening and not enrolled in this study. Daily living abilities were evaluated using the Activities of Daily Living (ADL) scale (total score 0–100; 100 indicates independence; <60 indicates impairment) and the Functional Activities Questionnaire (FAQ; ≥9 indicates significant functional dependence). All scales were administered by trained research personnel following standardized protocols.

### 2.3. Objective Evaluation of Sleep via Overnight PSG

None of the participants in the OSA or COMISA groups were receiving continuous positive airway pressure (CPAP) therapy at the time of enrollment, and CPAP was not used during the PSG recording night. All participants underwent overnight (>seven hours) PSG using a Philips Alice6 LDxN system (Philips Respironics, United States) on the same night following completion of questionnaires. To minimize the potential first-night effect, participants were instructed to wear their own sleepwear and bring personal items they typically use during sleep (such as pillows and blankets). They were also asked to avoid napping for more than 30 min during the daytime prior to the PSG night. Recordings included electroencephalography (EEG), electrocardiography (ECG), oxygen saturation, and other physiological parameters. EEG was recorded using six electrodes (F3, F4, C3, C4, O1, and O2) with mastoid references (M1 and M2). Bilateral anterior tibialis electromyography (EMG) was also recorded to monitor periodic limb movements. PSG measures included AHI, arousal index (ArI), sleep efficiency (SE), total sleep time (TST), sleep onset latency (SOL), wake after sleep onset (WASO), and other regular parameters. All PSG recordings were manually analyzed by certified sleep technologists at the Sleep Center according to the American Academy of Sleep Medicine (AASM) Scoring Manual Version 3.0, and the final reports were verified by sleep medicine physicians.

### 2.4. MRI Data Acquisition

Participants underwent MRI data acquisition using a 3.0-Tesla MR system (Prisma, Siemens Medical Solutions, Inc., Erlangen, Germany). Voxel-based morphometry (VBM) analysis was performed on the structural MRI (sMRI) data to examine between-group differences in gray matter density (GMD). Preprocessing of sMRI data was conducted using Statistical Parametric Mapping 12 (SPM12) and the CAT12.8.2 toolbox on the MATLAB 2022b platform, including segmentation, smoothing, and calculation of total intracranial volume (TIV) and gray matter volume (GMV). Additionally, rs-fMRI data were preprocessed using RESTplus v1.30 within MATLAB 2022b. Preprocessing steps included conversion from DICOM to NIFTI format, removal of the first 10 time points, slice timing correction, realignment, spatial normalization, smoothing, detrending, nuisance covariates regression, and filtering. Specific preprocessing steps varied slightly depending on the subsequent analysis. Following preprocessing, we computed amplitude of low-frequency fluctuation (ALFF), fractional ALFF (fALFF), regional homogeneity (ReHo), degree centrality (DC), functional connectivity density (FCD), and functional connectivity (FC). MRI acquisition parameters and analysis software are provided in Table S1.

### 2.5. Statistical Analysis

Statistical analysis was performed using SPSS 27.0. Normally distributed continuous data are presented as mean ± standard deviation (X ± SD) and were analyzed by one-way analysis of variance (ANOVA) with Bonferroni post hoc analysis. Non-normally distributed or heteroscedastic data are expressed as median (interquartile range) [M (P25, P75)] and were analyzed using the Kruskal–Wallis H test, with Dunn’s post hoc pairwise comparisons and Bonferroni correction. Categorical variables are described as frequencies and percentages (*n*, %) and were analyzed by the chi-square test, with Fisher’s exact test applied when expected frequencies were <5, and post hoc comparisons were conducted using the partition of chi-square method (α’ = 0.008). Spearman correlation was used to assess relationships among demographic, scale, PSG, and MRI data. Multiple linear regression was employed to explore the association between nocturnal awakenings and brain functional activity. All tests were two-tailed, with *p* < 0.05 considered statistically significant.

In MATLAB R2022b, one-way ANOVA was performed in SPM12 to assess differences in GMD among the four groups, with age, sex, years of education, recent regular use of hypnotic medications, and total intracranial volume (TIV) included as covariates. Similarly, one-way ANOVA was used to evaluate ALFF, fALFF, ReHo, FC, DC, and FCD differences in SPM12, with age, sex, years of education, and recent insomnia medication use as covariates. Brain regions were defined using the Automated Anatomical Labeling 116 (AAL116) atlas. Gaussian random field (GRF) correction (cluster-level *p* < 0.05) was applied using RESTplus (an improved toolkit for resting-state functional MRI data processing, version 1.30). Statistically significant clusters were saved via SPM12, and their values were extracted using RESTplus v1.30 for further statistical analysis. After extraction, further ANOVA with post hoc Bonferroni comparisons or Kruskal–Wallis H tests with Dunn–Bonferroni correction were conducted in SPSS 27.0 (α = 0.05). Finally, xjview version 9.6 (a MATLAB-based toolbox) and MRIcroGL version 1.2.20220720 were used to organize the data and visualize the significant clusters.

## 3. Results

### 3.1. Demographic and Clinical Characteristics

**Figure 1:**
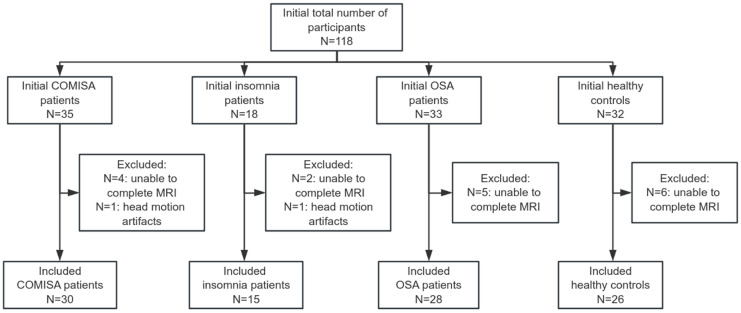
Flowchart of participant selection. COMISA, comorbid insomnia and sleep apnea; OSA, obstructive sleep apnea.

A total of 118 participants were initially recruited for this study. Two participants were excluded due to head motion artifacts during MRI, and another 17 were unable to complete the MRI examination. In total, 99 participants (mean age 43.42 years, 55.6% male) were included in the final analysis, comprising 30 with COMISA, 15 with insomnia alone, 28 with OSA alone, and 26 healthy controls. No clinically significant limb movement disorders, including restless legs syndrome or periodic limb movements, were identified in any participant based on PSG recordings and clinical assessment. A flow diagram of the study protocol is shown in Figure 1.

The demographic and clinical characteristics of the participants are summarized in Table 1. Significant differences among the four groups were observed in age, gender, body mass index (BMI), neck and waist circumferences, years of education, recent insomnia medication use, history of alcohol intake, and rates of comorbid hypertension and hyperlipidemia (all *p* < 0.05). Although there was an overall difference in alcohol intake among the groups, post hoc comparisons did not reveal significant pairwise differences. Patients with COMISA had higher BMI and neck and waist circumferences than those with insomnia (*p* = 0.015; *p* = 0.048; *p* < 0.001), but were similar to the OSA group (*p* = 1.000; *p* = 0.645; *p* = 1.000). Comorbid hypertension was higher in the COMISA group than in the insomnia group (*p* = 0.001) and healthy controls (*p* < 0.001). Comorbid hyperlipidemia was also higher in the COMISA group than in the healthy controls (*p* = 0.006).

### 3.2. Subjective and Objective Assessments of Sleep and Daytime Function

As shown in Table 1, significant differences were observed in the AIS, ISI, PSQI, STOP-Bang, ESS, and FSS scores among the four groups (all *p* < 0.05). Patients with COMISA had a higher STOP-Bang score than patients with insomnia (*p* = 0.005) and higher AIS, ISI, and PSQI scores than patients with OSA (*p* < 0.001; *p* < 0.001; *p* < 0.001). Regarding PSG parameters, ArI, AHI, nocturnal minimum and mean oxygen saturation, TST, SOL, and percentage of non-rapid eye movement sleep stage 1 (N1%) differed significantly among the four groups (all *p* < 0.05, Table 1). Post hoc analysis with correction showed no significant pairwise differences in TST or SOL. The COMISA group exhibited higher ArI and AHI and lower nocturnal minimum oxygen saturation than the insomnia (*p* = 0.001; *p* < 0.001; *p* = 0.001) and control groups (*p* = 0.001; *p* < 0.001; *p* < 0.001), but did not differ from the OSA group (*p* = 1.000; *p* = 1.000; *p* = 1.000). Additionally, patients with COMISA had a higher proportion of light sleep (N1%) than healthy controls (*p* < 0.001). To evaluate the potential influence of recent hypnotic use, we performed a sensitivity analysis excluding the 10 participants who reported taking such medications. As shown in Table S2, the overall pattern of group differences in AHI and ArI remained unchanged. The overall difference in SOL among the groups remained significant (*p* = 0.034); however, the previously observed group difference in TST was no longer significant after exclusion. All other findings were consistent with the main analysis.

### 3.3. MRI Findings

#### 3.3.1. Structural MRI Results

VBM analysis revealed no significant differences in GMD among the four groups at a statistical threshold of *p* < 0.001 (all *p* > 0.479).

#### 3.3.2. Functional MRI Results

**Figure 2:**
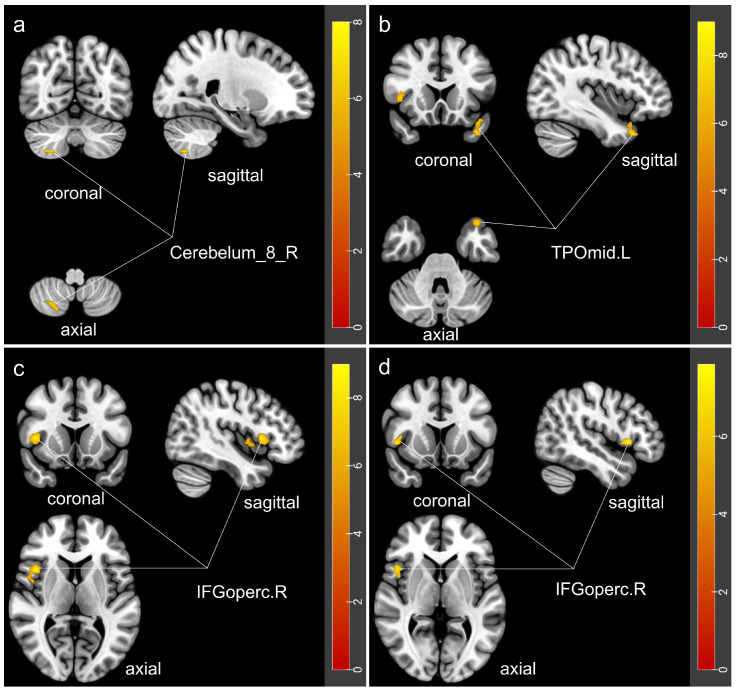
Group differences in regional functional metrics. (**a**) fALFF differences in the Cerebelum_8_R; (**b**) DC differences in the TPOmid.L; (**c**) DC differences in the IFGoperc.R; and (**d**) FCD differences in the IFGoperc.R. Statistical threshold was set at voxel-level *p* < 0.001, and cluster-level *p* < 0.05 with GRF correction. Color bars indicate F-values. Cerebelum_8_R, the right cerebellar lobule VIII; TPOmid.L, the left temporal pole of the middle temporal gyrus; IFGoperc.R, the right inferior frontal gyrus, opercular part.

Significant between-group differences were observed in fALFF, DC, and FCD in specific brain regions among the four groups (GRF-corrected, voxel-level *p* < 0.001, cluster-level *p* < 0.05), while no significant differences were found in ALFF or ReHo (Table 2; Figure 2). Detailed results of post hoc pairwise comparisons are shown in Table S3. In the COMISA group, the fALFF value in the right cerebellar lobule VIII (Cerebelum_8_R) was lower than that in the insomnia group (*p* = 0.005) and higher than that in the OSA group (*p* = 0.005). The DC value in the left temporal pole of the middle temporal gyrus (TPOmid.L) was significantly lower than that in the OSA group but comparable to that in the insomnia group (*p* < 0.001; *p* = 1.000). Furthermore, the COMISA group exhibited higher DC values in the right inferior frontal gyrus, opercular part (IFGoperc.R) than did the insomnia and OSA groups (*p* < 0.001; *p* = 0.026), whereas the FCD value in this region was lower than that in the OSA group (*p* = 0.001), with no significant difference compared to the insomnia group (*p* = 1.000).

**Figure 3:**
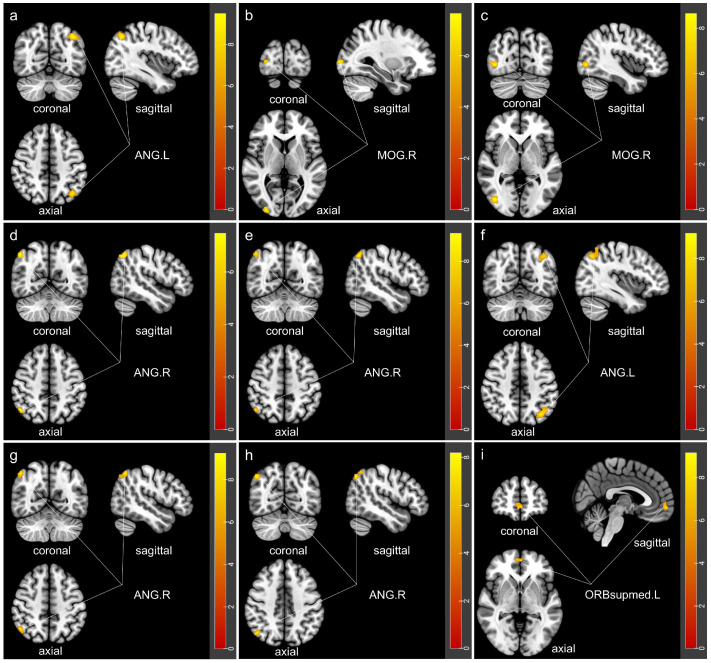
Brain regions showing significant group differences in functional connectivity. (**a**–**i**) Brain regions with significant between-group differences in functional connectivity (FC), corresponding to the nine seed-based FC analyses presented in Table 3. Statistical threshold was set at voxel-level *p* < 0.001 and cluster-level *p* < 0.05 with GRF correction. Color bars indicate F-values. ANG.L, left angular gyrus; MOG.R, right middle occipital gyrus; ANG.R, right angular gyrus; ORBsupmed.L, the left medial orbital superior frontal gyrus.

Based on the results of the fALFF, DC, and FCD analyses, as well as the involvement of arousal-related brain regions, the frontal lobe, temporal lobe, cerebellum, and parts of the limbic system were selected as seed regions for the FC analysis. While no significant differences in FC between the Cerebelum_8_R and other brain regions were found, significant between-group differences were identified in nine other brain regions after GRF correction (Table 3; Figure 3), with detailed post hoc test results in Table S3. In the frontal lobe, compared with the insomnia group, the COMISA group showed increased FC between the right dorsolateral superior frontal gyrus (SFGdor.R) and left angular gyrus (ANG.L), between the right middle frontal gyrus (MFG.R) and right middle occipital gyrus (MOG.R), and between the IFGoperc.R and MOG.R (*p* < 0.001; *p* = 0.041; *p* = 0.016). In addition, compared with the OSA group, the COMISA group exhibited increased FC between SFGdor.R and ANG.L (*p* = 0.018). In the temporal regions, FC between the right fusiform gyrus (FFG.R) and the right angular gyrus (ANG.R), the left middle temporal gyrus (MTG.L) and ANG.R, and the right middle temporal gyrus (MTG.R) and ANG.L was significantly higher in the COMISA group than in the insomnia group (*p* < 0.001; *p* = 0.001; *p* < 0.001). Within the cerebellum, the COMISA group showed stronger FC between the left cerebellar lobules IV–V (Cerebelum_4_5_L) and ANG.R than did the insomnia and control groups (*p* < 0.001; *p* = 0.002), and between the right cerebellar lobules IV–V (Cerebelum_4_5_R) and ANG.R relative to those in the insomnia, OSA, and control groups (*p* < 0.001; *p* = 0.015; *p* = 0.003). In contrast, the FC between the left cerebellar lobule IX (Cerebelum_9_L) and the left medial orbital superior frontal gyrus (ORBsupmed.L) was significantly lower in the COMISA group than in the insomnia group (*p* = 0.015). No significant between-group differences were detected in FC involving the cingulate gyrus (AAL31-36), hippocampus (AAL37-38), or amygdala (AAL41-42) (*p* > 0.111, *p* > 0.245, *p* > 0.466).

#### 3.3.3. Correlation and Regression Analyses

Given the role of nocturnal awakenings and fragmentation in daytime impairment, the arousal index (ArI) was used as the dependent variable, and our analysis confirmed that the COMISA group had a higher ArI than the insomnia-alone group. ArI showed positive correlations with BMI, history of hypertension, ESS score, AHI, and several FC and DC measures, while negative correlations were observed with gender, years of education, and the fALFF value of the Cerebelum_8_R. Full correlation coefficients and *p*-values are presented in Table 4. Multiple linear regression analysis, incorporating all brain regions with functional abnormalities, revealed that only the fALFF value of Cerebelum_8_R had a significant independent association with ArI. As presented in Table 5, in Model 1, which was adjusted for demographic characteristics, history of hypnotic medication use, and cardiometabolic risk factors, decreased functional activity in this cerebellar region was associated with more frequent nocturnal awakenings (β = −0.268, *p* = 0.010). This association was attenuated and became non-significant after adding AHI in Model 2 (β = −0.155, *p* = 0.098). Importantly, however, the association remained significant in Model 3, in which ISI score and SE were added to the covariates in Model 1 (β = −0.283, *p* = 0.011).

## 4. Discussion

This study investigated the relationship between nocturnal arousal and brain function in patients with COMISA. Compared to patients with insomnia alone and healthy controls, those with COMISA exhibited a higher nocturnal arousal index (ArI). Neuroimaging analyses revealed that, although no significant group differences were found in gray matter density, patients with COMISA showed distinct alterations in both regional activity and functional connectivity involving multiple brain regions, including the frontal lobe, temporal lobe, and cerebellum. The pattern and magnitude of these alterations differed from those observed in patients with insomnia alone or OSA alone. Further correlation analyses revealed that increased nocturnal awakenings in patients with COMISA were independently associated with reduced local functional activity in the right cerebellar lobule VIII (Cerebelum_8_R). This association was influenced by AHI but remained independent of insomnia severity and sleep efficiency.

The cerebellum has increasingly been recognized as more than a motor structure and may contribute to sleep–awake regulation and arousal processes [25,26]. It receives inputs from neuromodulatory systems related to arousal regulation and projects to multiple brain regions involved in wakefulness and sleep control, supporting a modulatory role in arousal regulation [25,27]. In the present study, patients with COMISA exhibited altered local activity in the right cerebellar lobule VIII (Cerebelum_8_R), with fALFF values significantly lower than those in the insomnia group but higher than those in the OSA group. Additionally, functional connectivity between the left cerebellar lobule IX (Cerebelum_9_L) and the left medial orbital superior frontal gyrus (ORBsupmed.L) was significantly increased in the insomnia group compared to the COMISA and OSA groups, while no difference was found between COMISA and OSA. The medial orbital superior frontal gyrus is involved in decision-making, attention, and autonomic regulation, and the cerebellar–frontal circuit is critical for regulating cognition and maintaining sleep [28,29]. The increased connectivity in the insomnia group highlights a potential role of this circuit in insomnia-related pathophysiology, whereas its absence in COMISA may reflect that this regulatory mechanism is affected by OSA-related factors. The altered activity in cerebellar lobule VIII and the altered connectivity between cerebellar lobule IX and prefrontal regions illustrate the involvement of posterior cerebellar subregions in mechanisms underlying arousal and frequent awakenings. These findings suggest that cerebello-cortical circuits may be involved in the regulation of nocturnal arousal and sleep fragmentation, with different posterior cerebellar subregions contributing through distinct neurophysiological pathways.

Notably, reduced fALFF in the right cerebellar lobule VIII was independently associated with increased nocturnal awakenings (ArI) in patients with COMISA (β = −0.268, *p* = 0.010), after adjusting for demographic factors, medication use, and cardiovascular risk factors. This association remained significant when further adjusting for insomnia severity and sleep efficiency (β = −0.283, *p* = 0.011), suggesting that the relationship between cerebellar dysfunction and nocturnal arousal is independent of subjective insomnia symptoms. However, when AHI was added to the model, the association was no longer significant (β = −0.155, *p* = 0.098), indicating that sleep-disordered breathing may mediate this relationship. Previous studies have found abnormal functional connectivity between the right cerebellum and default mode network (DMN) nodes (angular gyrus and precuneus) in patients with OSA, which showed negative correlations with arousal index and were also associated with nocturnal hypoxia [30,31]. These findings suggest that the cerebellum may be susceptible to hypoxia-related injury in OSA, potentially contributing to disrupted arousal regulation. The cerebellar cortex has a relatively uniform architecture. Hypoxic injury would be expected to affect multiple subregions. However, we observed selective involvement of lobule VIII, suggesting differential vulnerability to hypoxia within the cerebellum. This selectivity may reflect functional specialization within the cerebellum. The cerebellum has been proposed to act as a predictive controller that constructs internal models of sensorimotor and autonomic states [32]. During sleep, these internal models may help maintain stability in response to respiratory or sensory perturbations. Disruption of this process may be associated with increased arousal responses to recurrent respiratory events and sleep fragmentation. This may be particularly relevant in patients with OSA and COMISA, in whom recurrent hypoxia and respiratory events challenge sleep stability. Furthermore, the cerebellum interacts closely with the thalamus via cerebello-thalamo-cortical circuits in sleep–wake regulation [33,34]. The thalamus exhibits diverse electrophysiological patterns, including bursting and oscillatory rhythms, that shape and synchronize cortical activity, processes that are particularly relevant during sleep [33,34,35]. Alterations in this circuit may amplify arousal responses to respiratory events and contribute to fragmentation. Future studies combining high-resolution imaging and electrophysiological measures are needed to clarify the role of this pathway.

To our knowledge, this is the first study to illustrate the differences in arousal among patients with insomnia, OSA, and COMISA and to explore the association between ArI and rs-fMRI. Both objective and subjective sleep assessments were included, and the potential influences of medication-treated insomnia, cognitive function, and emotional status were carefully considered. Our findings extend the existing neuroimaging literature in several ways. As noted above, prior studies in patients with OSA have reported abnormal functional connectivity between the cerebellum and default mode network, which was associated with nocturnal hypoxia and arousal index [30,31]. Our observation of selective involvement of the right cerebellar lobule VIII in COMISA aligns with the concept of functional specialization within the cerebellum and suggests that distinct subregions may contribute differently to sleep stability. Moreover, our results in patients with COMISA differ from those in patients with insomnia alone, where cerebellar–frontal connectivity was increased, supporting the notion that COMISA may have unique neuroimaging characteristics beyond those of either condition alone. Although studies specifically examining cerebellar function in COMISA are lacking, our findings provide the first evidence linking Cerebelum_8_R activity to nocturnal arousal in this population, thereby filling a gap in the literature.

In this study, patients with COMISA exhibited a significantly higher arousal index than those with insomnia alone, suggesting an additive effect of sleep apnea on sleep instability in COMISA. ArI showed a significant positive correlation with ESS score, indicating that nocturnal sleep fragmentation may contribute to daytime functional impairment. Notably, reduced functional activity in the right cerebellar lobule VIII was independently associated with sleep fragmentation, and this association remained significant after controlling for insomnia severity. This finding suggests that Cerebelum_8_R may play a critical role in the central regulation of sleep stability in COMISA and could serve as a potential neuroimaging-informed target for adjunctive therapeutic strategies, complementing conventional treatments such as cognitive behavioral therapy for insomnia or continuous positive airway pressure (CPAP). Emerging evidence supports the feasibility of cerebellar neuromodulation, including transcranial magnetic stimulation and transcranial direct current stimulation, as a means of influencing clinical outcomes in neurological disorders [36,37]. Early studies have demonstrated that non-invasive cerebellar stimulation can modulate cerebellar processing and related behavior, while the cerebellum exhibited unique plasticity mechanisms with extensive connections to neocortical areas [36]. More recently, a comprehensive review proposed that the cerebellum, long regarded as a relatively overlooked structure in neurodegenerative diseases, may represent a promising neuromodulatory target [37]. Our identification of Cerebelum_8_R as a key node associated with arousal may be consistent with this concept, suggesting that this subregion could represent a potential target for adjunctive interventions in COMISA. In particular, multi-night polysomnography and CPAP intervention studies may help clarify the temporal relationship between Cerebelum_8_R functional changes and respiratory events, and determine whether normalization of cerebellar activity reduces nocturnal arousal. In addition, longitudinal studies are needed to further explore these mechanisms. However, given the cross-sectional nature of this study, causal relationships cannot be established, and this hypothesis should be further validated in future longitudinal or interventional research.

Beyond cerebellar-related abnormalities, additional functional connectivity alterations were observed among cortical regions implicated in arousal regulation and cognitive–sensory processing. Notably, the functional connectivity between the MFG.R and MOG.R, as well as between the IFGoperc.R and MOG.R, was positively correlated with the arousal index. Consistent with previous research, this extensive hyperconnectivity may reflect a state of sustained hyperarousal during sleep, thereby disrupting nocturnal sleep [38]. In addition, the angular gyrus (ANG), a key node in the DMN, plays an important role in sleep-related neural activity and sleep–wake regulation [39,40,41]. Prior studies have shown increased neural activity in the ANG and other DMN regions in patients with chronic insomnia compared with controls [42], supporting the notion that persistent DMN activation may interfere with normal sleep maintenance. Together, these findings suggest that hyperactive sensory-related circuits and DMN-related networks may contribute to increased nocturnal awakenings in patients with COMISA.

The right inferior frontal gyrus, opercular part (IFGoperc.R), a region critically involved in cognitive control and response inhibition, plays a central role in suppressing inappropriate behavior and neural responses [43]. In the present study, patients with COMISA had higher DC but lower FCD in the IFGoperc.R, a pattern that may reflect a compensatory mechanism for maintaining information integration under conditions of network inefficiency [44,45]. Such alterations in frontal control networks may further exacerbate vulnerability to arousal by impairing the regulation of sleep-related neural processes.

In addition to the neuroimaging findings, our study also revealed important clinical characteristics of patients with COMISA. Our findings indicated that patients with COMISA exhibited more severe insomnia symptoms and poorer daytime function, whereas most PSG data, including SE, WASO, and percentage of non-rapid eye movement sleep stage 3 (N3%), did not show significant differences. There is often a discrepancy between subjective and objective sleep in COMISA, reflecting an overestimation of sleep disturbance [46] and highlighting the complex relationship between perceived and measured sleep quality [6]. In our study, patients with COMISA exhibited a higher arousal index compared with those in the insomnia group. Previous research, however, has reported no significant differences in the total number of nocturnal awakenings or arousal index among patients with COMISA, insomnia, and OSA [47]. These observations suggest that increased nocturnal arousals in COMISA are not solely caused by respiratory effort or insomnia and may depend on patient subtype. In our study, patients with moderate-to-severe OSA and mild-to-moderate insomnia appeared to experience more frequent nocturnal awakenings, likely because OSA exacerbates underlying insomnia. This pattern is consistent with the mechanisms described in OSA [48,49].

An important negative finding of this study is that no significant between-group differences in functional connectivity were observed in limbic system regions such as the cingulate gyrus, hippocampus, or amygdala. This finding differs from those of some previous studies on insomnia and OSA that reported limbic alterations [50,51,52,53]. One possible explanation is the strict exclusion criteria used in this study, as participants with mood disorders or cognitive impairment were excluded. The absence of these common comorbidities may partly account for the lack of limbic findings in our sample.

However, this study has several limitations. First, the relatively small sample size, particularly for the insomnia-only group, limited the statistical power for subgroup analyses and may have influenced the findings. However, this sample size is consistent with those reported in previous neuroimaging studies of insomnia disorders [54]. As noted by Aquino et al. [55], small sample sizes represent a recognized challenge in this field. It should be noted that the limited number of eligible participants in the insomnia-only group was partly due to the strict inclusion and exclusion criteria, including the exclusion of psychiatric disorders, cognitive impairment, severe medical conditions, and individuals who did not undergo an MRI. Second, sleep architecture was assessed using a single night of polysomnography without an adaptation night, which may introduce first-night effects and potentially underestimate the variability of habitual arousal. Therefore, future studies using multi-night PSG would help better capture sleep architecture and arousal patterns. Of note, arousal-related parameters have been reported to demonstrate high intra-individual stability across nights in healthy adults [56], offering indirect support for the reliability of our single-night arousal index measurements. Third, the cross-sectional design does not allow causal inference of sleep disturbances and neuroimaging findings, including the temporal relationship between Cerebelum_8_R functional changes and respiratory events. Longitudinal or interventional studies (such as CPAP) are needed to determine whether alterations in cerebellar activity precede or follow respiratory events. Finally, it should be noted that the significance of group differences in TST was sensitive to the exclusion of participants with recent hypnotic use, which may partly reflect reduced statistical power given the small size; therefore, the TST finding should be interpreted with caution.

## 5. Conclusions

In this study, patients with COMISA showed a higher arousal index compared with those with insomnia alone. Reduced functional activity in Cerebelum_8_R was independently associated with sleep fragmentation, and this association was independent of insomnia severity. These findings suggest that Cerebelum_8_R may be involved in the central regulation of sleep instability in COMISA and may serve as a potential adjunctive target alongside conventional therapies.

## Figures and Tables

**Table 1:** Demographic and Clinical Characteristics, Questionnaire Scores, and Polysomnographic Data of All Participants.

	COMISA (*n* = 30)	Insomnia (*n* = 15)	OSA (*n* = 28)	Control (*n* = 26)	*F*/*χ*^2^ Value	*p* Value
Age (year)	48.07 ± 10.82 *	43.27 ± 13.12	44.93 ± 11.59 *	36.54 ± 9.39 ^†§^	5.291	0.002
Gender (Male, %)	21.00 (70.0%) *	5.00 (33.3%) ^§^	21.00 (75.0%) ^‡^*	8.00 (30.8%) ^†§^	16.292	0.001
BMI (kg/m^2^)	25.90 (23.65, 30.10) ^‡^*	21.30 (20.40, 24.20) ^†§^	27.75 (24.30, 31.13) ^‡^*	23.35 (21.00, 24.60) ^†§^	26.831	<0.001
Neck circumference (cm)	38.00 (35.75, 41.00) ^‡^	34.00 (33.00, 36.00) ^†§^	41.00 (36.13, 42.75) ^‡^*	34.50 (33.00, 36.25) ^§^	24.698	<0.001
Waist circumference (cm)	94.23 ± 12.63 ^‡^*	80.00 ± 9.82 ^†§^	96.95 ± 9.94 ^‡^*	81.69 ± 8.72 ^†§^	15.511	<0.001
Years of education (year)	12.00 (9.00, 15.00) *	12.00 (9.00, 16.00) *	14.00 (9.50, 15.75) *	16.00 (15.75, 17.25) ^†‡§^	23.409	<0.001
Recent insomnia medication use (*n*, %)	6.00 (20.00%)	4.00 (26.70%) ^§^*	0 (0%) ^‡^	0 (0%) ^‡^	13.307	<0.001
Current smoker (*n*, %)	5.00 (16.70%)	1.00 (6.70%)	8.00 (28.60%)	2.00 (7.70%)	4.900	0.176
Alcohol intake (*n*, %)	6.00 (20.00%)	0 (0%)	5.00 (17.90%)	0 (0%)	8.683	0.019
Hypertension (*n*, %)	14.00 (46.70%) ^‡^*	0 (0%) ^†§^	13.00 (46.40%) ^‡^*	1.00 (3.80%) ^†§^	23.114	<0.001
Diabetes (*n*, %)	3.00 (10.00%)	0 (0%)	1.00 (3.60%)	0 (0%)	3.132	0.311
Hyperlipidemia (*n*, %)	10.00 (33.30%) *	1.00 (6.70%)	7.00 (25.00%)	1.00 (3.80%) ^†^	9.878	0.015
Family history of OSA (*n*, %)	19.00 (63.30%)	10.00 (66.70%)	21.00 (75.00%)	14.00 (53.80%)	2.690	0.442
AIS score	11.00 (9.75, 13.00) ^§^*	10.00 (8.00, 11.00) ^§^*	3.00 (1.25, 4.00) ^†‡^	0.50 (0, 1.00) ^†‡^	79.312	<0.001
ISI score	17.50 (16.75, 19.25) ^§^*	17.00 (16.00, 19.00) ^§^*	3.50 (1.00, 5.00) ^†‡^	0 (0, 0) ^†‡^	81.318	<0.001
PSQI score	13.50 (11.00, 16.50) ^§^*	12.00 (9.00, 15.00) ^§^*	5.00 (3.25, 7.00) ^†‡^*	1.00 (0, 2.00) ^†‡§^	79.266	<0.001
STOP Bang score	5.00 (2.00, 5.00) ^‡^*	1.00 (0, 2.00) ^†§^	4.50 (3.00, 6.00) ^‡^*	0 (0, 1.00) ^†§^	50.513	<0.001
ESS score	5.00 (3.00, 12.00) *	1.00 (0, 6.00)	6.00 (2.25, 12.75) *	0 (0, 2.00) ^†§^	34.299	<0.001
FSS score	32.00 (9.00, 52.50) *	21.00 (9.00, 37.00) *	9.00 (9.00, 47.00) *	9.00 (9.00, 9.00) ^†‡§^	26.966	<0.001
ArI (events/h)	17.75 (12.98, 28.45) ^‡^*	8.80 (5.70, 11.00) ^†§^	18.40 (15.15, 29.58) ^‡^*	9.40 (6.50, 15.80) ^†§^	33.157	<0.001
AHI (events/h)	27.05 (9.75, 39.45) ^‡^*	2.10 (0.70, 3.60) ^†§^	35.00 (14.88, 61.48) ^‡^*	1.40 (0.88, 3.50) ^†§^	72.173	<0.001
Min SpO2 (%)	84.00 (77.75, 89.25) ^‡^*	92.00 (90.00, 93.00) ^†§^	83.00 (67.25, 87.50) ^‡^*	93.00 (92.00, 94.00) ^†§^	57.820	<0.001
Mean SpO2 (%)	95.00 (94.00, 96.00) ^‡^*	97.00 (96.00, 97.00) ^†§^	95.00 (94.00, 96.00) ^‡^*	96.50 (96.00, 97.00) ^†§^	23.964	<0.001
SE (%)	80.95 (73.33, 91.30)	76.50 (66.80, 89.80)	85.45 (76.90, 93.70)	84.10 (79.93, 92.55)	6.419	0.093
TST (min)	379.55 (330.75, 429.73)	367.00 (314.50, 428.00)	428.00 (365.50, 460.38)	396.25 (381.70, 433.88)	9.083	0.028
SOL (min)	13.30 (7.48, 28.63)	18.50 (9.50, 44.20)	7.55 (3.80, 15.18)	14.75 (8.25, 27.75)	9.202	0.027
WASO (min)	54.75 (25.63, 96.50)	63.50 (31.00, 115.50)	46.25 (22.75, 79.88)	39.25 (17.50, 71.63)	1.997	0.573
REM latency (min)	112.75 (85.63, 164.00)	93.50 (71.00, 127.50)	100.75 (89.38, 140.13)	86.00 (70.25, 146.25)	4.076	0.253
NREM%	84.12 + 6.09	82.23 + 5.85	83.39 + 5.91	82.67 + 4.65	0.498	0.684
N1%	18.75 (12.95, 25.13) *	11.50 (8.80, 20.10)	14.90 (11.60, 21.93) *	9.65 (7.58, 13.55) ^†§^	20.751	<0.001
N2%	52.44 ± 7.00	52.29 + 7.29	53.54 ± 7.97	56.24 ± 7.45	1.480	0.225
N3%	12.08 ± 7.49	15.47 ± 8.27	11.97 + 6.52	15.53 ± 6.66	1.910	0.133

COMISA, comorbid insomnia and sleep apnea; OSA, obstructive sleep apnea; BMI, body mass index; AIS, Athens Insomnia Scale; ISI, Insomnia Severity Index; PSQI, Pittsburgh Sleep Quality Index; ESS, The Epworth Sleeping Scale; FSS, Fatigue Severity Scale; ArI, Arousal index; AHI, Apnea Hypopnea Index; SE, Sleep Efficiency; TST, Total Sleep Time; SOL, Sleep Onset Latency; WASO, Wake After Sleep Onset; NREM, Non-Rapid Eye Movement. Post hoc pairwise comparisons (*p* < 0.05): * vs. controls; ^†^ vs. COMISA group; ^‡^ vs. insomnia group; ^§^ vs. OSA group.

**Table 2:** Characteristics of the Clusters with Significant Between-Group Differences in fALFF, DC, and FCD.

Ways	Cluster	Cluster Size	Brain Regions	Peak MNI Coordinate			Peak *F* Value	Post Hoc Comparisons
				x	y	z		
fALFF	Cluster 1	14	Cerebelum_8_R	24	−69	−54	8.37	COMISA < Insomnia, COMISA > OSA, Insomnia > OSA, Control > OSA
DC	Cluster 1	25	TPOmid.L	−42	18	−24	8.22	COMISA < OSA, Insomnia < OSA, Control < OSA
	Cluster 2	63	IFGoperc.R	42	0	0	9.40	COMISA > Insomnia, COMISA > OSA
FCD	Cluster 1	26	IFGoperc.R	45	15	0	8.09	COMISA < OSA, Control < OSA

MNI, Montreal Neurological Institute; COMISA, comorbid insomnia and sleep apnea; OSA, obstructive sleep apnea; fALFF, Fractional Amplitude of Low-Frequency Fluctuations; DC, Degree Centrality; FCD, Functional Connectivity Density; Oper, opercular; Mid, middle; R, right; L, left; TPO, Temporal Pole; IFG, Inferior Frontal Gyrus.

**Table 3:** Characteristics of the Clusters with Significant Between-Group Differences in Functional Connectivity.

ROI	Cluster	Cluster Size	Connected Region	Peak MNI Coordinate			Peak F Value	Post Hoc Comparisons
				x	y	z		
SFGdor.R	Cluster 1	59	ANG.L	−39	−66	45	9.70	COMISA > Insomnia & OSA, Insomnia < Control
MFG.R	Cluster 1	29	MOG.R	33	−96	3	7.84	COMISA > Insomnia & Control, OSA > Insomnia & Control
IFGoperc.R	Cluster 1	33	MOG.R	42	−75	0	8.80	COMISA > Insomnia, OSA > Insomnia & Control
FFG.R	Cluster 1	28	ANG.R	48	−66	45	7.64	COMISA > Insomnia & Control, OSA > Insomnia
MTG.L	Cluster 1	29	ANG.R	48	−63	48	9.61	COMISA > Insomnia & Control, OSA > Insomnia & Control
MTG.R	Cluster 1	99	ANG.L	−36	−69	48	9.31	Insomnia < COMISA & OSA
Cerebelum_4_5_L	Cluster 1	33	ANG.R	48	−60	51	9.51	COMISA > Insomnia & Control
Cerebelum_4_5_R	Cluster 1	32	ANG.R	48	−69	45	8.42	COMISA > Insomnia & OSA & Control
Cerebelum_9_L	Cluster 1	28	ORBsupmed.L	0	57	−6	9.45	Insomnia > COMISA & OSA

MNI, Montreal Neurological Institute; COMISA, comorbid insomnia and sleep apnea; OSA, obstructive sleep apnea; Sup, superior; Oper, opercular; Mid, middle; R, right; L, left; ANG, Angular Gyrus; FFG, Fusiform Gyrus; IFG, Inferior Frontal Gyrus; MFG, Middle Frontal Gyrus; MOG, Middle Occipital Gyrus; MTG, Middle Temporal Gyrus; ORBmed, Medial Orbital Gyrus; SFG, Superior Frontal Gyrus; TPO, Temporal Pole. The nomenclature for cerebellar regions follows the AAL atlas and has been retained as provided.

**Table 4:** Spearman Correlation Analysis for the Total Sample.

PSG Parameter	Other Variables	*ρ* Value	*p* Value	Other Variables	*ρ* Value	*p* Value	Other Variables	*ρ* Value	*p* Value
ArI	Age	0.112	0.268	ESS	0.343	0.001	fALFF in Cerebelum_8_R	−0.409	<0.001
	Gender	−0.417	<0.001	STOP Bang	0.541	<0.001	DC in TPOmid.L	0.227	0.024
	BMI	0.355	<0.001	FSS	0.202	0.045	DC in IFGoperc.R	0.147	0.146
	Years of education	−0.242	0.016	AHI	0.658	<0.001	FCD in IFGoperc.R	0.152	0.132
	Recent insomnia medication use	−0.017	0.867	SE	0.017	0.864	FC between SFG.R and ANG.L	0.062	0.544
	Hypertension	0.358	<0.001	TST	0.100	0.327	FC between MFG.R and MOG.R	0.312	0.002
	Diabetes	0.070	0.491	SOL	−0.174	0.085	FC between IFGoperc.R and MOG.R	0.228	0.023
	Hyperlipidemia	0.145	0.152	WASO	0.170	0.093	FC between FFG.R and ANG.R	0.132	0.192
	Current smoker	0.326	0.001	REM latency	0.226	0.024	FC between MTG.L and ANG.R	0.154	0.129
	Alcohol intake	0.348	<0.001	NREM%	0.161	0.112	FC between MTG.R and ANG.L	0.124	0.220
	AIS	0.160	0.113	N1%	0.567	<0.001	FC between Cerebelum_4_5_L and ANG.R	0.238	0.018
	ISI	0.125	0.216	N2%	−0.103	0.311	FC between Cerebelum_4_5_R and ANG.R	0.150	0.139
	PSQI	0.178	0.077	N3%	−0.415	<0.001	FC between Cerebelum_9_L and ORBmed.L	−0.156	0.124

ArI, Arousal index; AIS, Athens Insomnia Scale; ISI, Insomnia Severity Index; PSQI, Pittsburgh Sleep Quality Index; ESS, The Epworth Sleeping Scale; FSS, Fatigue Severity Scale; AHI, Apnea Hypopnea Index; SE, Sleep Efficiency; TST, Total Sleep Time; SOL, Sleep Onset Latency; WASO, Wake After Sleep Onset; NREM, Non-Rapid Eye Movement; Oper, opercular; Mid, middle; R, right; L, left; fALFF, Fractional Amplitude of Low-Frequency Fluctuations; DC, Degree Centrality; FCD, Functional Connectivity Density; ANG, Angular Gyrus; FFG, Fusiform Gyrus; IFG, Inferior Frontal Gyrus; MFG, Middle Frontal Gyrus; MOG, Middle Occipital Gyrus; MTG, Middle Temporal Gyrus; ORBmed, Medial Orbital Gyrus; SFG, Superior Frontal Gyrus; TPO, Temporal Pole. The nomenclature for cerebellar regions follows the AAL atlas and has been retained as provided.

**Table 5:** Multiple Linear Regression Analysis of the Association Between Arousal Index and fALFF Values in the Cerebelum_8_R.

Covariates	B	SE	β	t	*p* Value	VIF	*F* Value	R^2^
Model 1: Age, Sex, BMI, Years of Education, History of Recent Regular Use of Hypnotics, History of Hypertension, History of Hyperlipidemia	−0.147	0.055	−0.268	−2.648	0.010	1.410	5.949	0.346
Model 2: Model 1 + AHI	−0.085	0.051	−0.155	−1.670	0.098	1.499	9.397	0.487
Model 3: Model 1 + ISI Score, Sleep Efficiency	−0.155	0.059	−0.283	−2.609	0.011	1.592	4.691	0.348

BMI, body mass index; AHI, Apnea Hypopnea Index; ISI, Insomnia Severity Index. B, unstandardized regression coefficient; SE, standard error; β, standardized regression coefficient; t, t-value; VIF, variance inflation factor; R^2^, coefficient of determination.

## Data Availability

The data presented in this study are available on request from the corresponding author due to privacy and ethical restrictions.

## Supplementary Materials

The following supporting information can be downloaded at: https://www.mdpi.com/article/10.3390/jcm15083080/s1. Table S1: Acquisition Parameters and Analysis Software of MRI. Table S2: Polysomnographic Parameters in Participants, Excluding Recent Hypnotic Medication Users. Table S3: Supplementary Post Hoc MRI Findings among the Four Groups.
