# Supplementary material for: Altered Cerebellar Spontaneous Activity and Its Association with Arousal Index in Comorbid Insomnia and Obstructive Sleep Apnea: A Resting-State fMRI Study
Source: J Clin Med. 2026 Apr 17;15(8):3080. doi: 10.3390/jcm15083080 (PMC13117985; doi:10.3390/jcm15083080)
Supplement: Supplementary file 1 [file jcm-15-03080-s001.zip › jcm-4235195-supplementary.pdf]

Table S1 Acquisition Parameters and Analysis Software of MRI

Structural MRI		Resting-state functional MRI (rs-fMRI)	
repetition time (TR)	3000ms	repetition time (TR)	1500ms
echo time (TE)	2.56ms	echo time (TE)	31ms
inversion time (TI)	1100ms	flip angle (FA)	70°
flip angle (FA)	7°	field of view (FOV)	211 × 211 mm^2
slice thickness	0.8 mm	matrix size	88 × 88
slices per slab	192	number of slices	60
field of view (FOV)	256 × 256 mm^2	slice thickness	2.4mm
matrix size	256 × 256	gap	0
voxel size	0.8 × 0.8 × 0.8 mm^3	voxel size	2.4 × 2.4 × 2.4 mm^3
total acquisition time	8 minutes and 35 seconds	per run	300 time points
		total scan duration	7 minutes and 40 seconds

Note: Structural MRI data were acquired using a 64-channel coil with a 3D T1-weighted magnetization-prepared rapid gradient-echo (3D T1WI MP-RAGE) sequence to obtain high-resolution anatomical images. Voxel-based morphometry (VBM) analysis was performed using the Statistical Parametric Mapping package (SPM12, implemented in MATLAB R2022b) to assess structural differences. rs-fMRI data were acquired using a blood oxygenation level-dependent (BOLD) gradient-echo echo-planar imaging (GE-EPI) sequence. rs-fMRI data were analyzed using RESTplus v1.30 and SPM12 within the MATLAB R2022b environment to examine between-group differences.

Table S2 Polysomnographic Parameters in Participants Excluding Recent Hypnotic Medication Users

	COMISA (n = 24)	Insomnia (n = 11)	OSA (n = 28)	Control (n = 26)	F /χ² value	P value
Arousal index (events/h)	18.90 (13.10, 28.55) ‡*	8.30 (5.70, 10.40) †§	18.40 (15.15, 29.58) ‡*	9.40 (6.50, 15.80) †§	34.232	**<0.001**
AHI (events/h)	30.65 (10.23, 50.70) ‡*	2.20 (0.70, 4.00) †§	35.00 (14.88, 61.48) ‡*	1.40 (0.88, 3.50) †§	64.545	**<0.001**
Min SpO_2 (%)	83.50 (71.00, 88.50) ‡*	91.00 (89.00, 95.00) †§	83.00 (67.25, 87.50) ‡*	93.00 (92.00, 94.00) †§	52.172	**<0.001**
Mean SpO_2 (%)	95.00 (94.00, 96.00) ‡*	97.00 (96.00, 98.00) †§	95.00 (94.00, 96.00) ‡*	96.50 (96.00, 97.00) †§	23.944	**<0.001**
SE (%)	81.15 (74.40, 92.10)	79.90 (68.00, 90.40)	85.45 (76.90, 93.70)	84.10 (79.93, 92.55)	3.148	0.369
TST (min)	382.30 (347.38, 434.18)	369.20 (332.30, 428.00)	428.00 (365.50, 460.38)	396.25 (381.70, 433.88)	5.480	0.140
SOL (min)	14.25 (8.18, 32.88)	18.50 (9.70, 38.00)	7.55 (3.80, 15.18)	14.75 (8.25, 27.75)	8.689	**0.034**
WASO (min)	54.75 (23.88, 93.38)	38.50 (7.50, 83.50)	46.25 (22.75, 79.88)	39.25 (17.50, 71.63)	1.002	0.801
REM latency (min)	107.75 (88.25, 165.00)	82.00 (67.50, 121.00)	100.75 (89.38, 140.13)	86.00 (70.25, 146.25)	5.036	0.169
NREM%	84.55 ± 6.02	81.66 ± 5.55	83.39 ± 5.91	82.67 ± 4.65	0.838	0.477
N1%	18.75 (14.45, 24.63) *	11.50 (7.90, 20.10)	14.90 (11.60, 21.93) *	9.65 (7.58, 13.55) †§	20.737	**<0.001**
N2%	52.10 ± 6.36	52.15 ± 7.53	53.54 ± 7.97	56.24 ± 7.45	1.578	0.201
N3%	12.91 ± 7.39	15.82 ± 9.49	11.97 ± 6.52	15.53 ± 6.66	1.511	0.218

Note: Post hoc pairwise comparisons (p<0.05): * vs. controls; † vs. COMISA group; ‡ vs. insomnia group; § vs. OSA group.

Abbreviations: COMISA, comorbid insomnia and sleep apnea; OSA, obstructive sleep apnea; ArI, Arousal index; AHI, Apnea Hypopnea Index; SE, Sleep Efficiency; TST, Total Sleep Time; SOL, Sleep Onset Latency; WASO, Wake After Sleep Onset; NREM, Non-Rapid Eye Movement; REM, Rapid Eye Movement.

Table S3 Supplementary Post-Hoc MRI Findings among the Four Groups

	COMISA (n = 30)	Insomnia (n = 15)	OSA (n = 28)	Control (n = 26)	F /χ² value	P value
fALFF in Cerebelum_8_R	−0.29 ± 0.36‡§	0.18 ± 0.55†§	−0.67 ± 0.49†‡*	−0.08 ± 0.34§	15.717	<0.001
DC in TPOmid.L	−0.05 (−0.18, 0.22) §	0.10 (−0.10, 0.27) §	0.42 (0.26, 0.55) †‡*	0 (−0.16, 0.20) §	26.172	<0.001
DC in IFGoperc.R	−0.05 (−0.1, 0.09) ‡§	−0.27 (−0.34, −0.17) †	−0.19 (−0.28, −0.02) †	−0.09 (−0.22, 0)	19.544	<0.001
FCD in IFGoperc.R	−0.06 (−0.16, 0.16) §	0.15 (−0.18, 0.30)	0.49 (0.07, 0.77) †*	0.05 (−0.25, 0.21) §	18.777	<0.001
FC between SFG.R and ANG.L	0.53 (0.38, 0.70) ‡§	0.26 (0.15, 0.36) †*	0.41 (0.27, 0.48) †	0.41 (0.28, 0.55) ‡	20.887	<0.001
FC between MFG.R and MOG.R	0.20 ± 0.18‡*	0.05 ± 0.15†§	0.24 ± 0.20‡*	0.02 ± 0.17†§	9.451	<0.001
FC between IFGoperc.R and MOG.R	0.20 ± 0.21‡	−0.01 ± 0.15†§	0.32 ± 0.21‡*	0.09 ± 0.23§	9.971	<0.001
FC between FFG.R and ANG.R	0.26 ± 0.18‡*	0 ± 0.15†§	0.15 ± 0.13‡	0.12 ± 0.16†	9.383	<0.001
FC between MTG.L and ANG.R	0.46 (0.36, 0.62) ‡*	0.23 (0.19, 0.33) †§	0.45 (0.3, 0.52) ‡*	0.31 (0.13, 0.39) †§	23.123	<0.001
FC between MTG.R and ANG.L	0.51 ± 0.23‡	0.19 ± 0.14†§	0.43 ± 0.16‡	0.37 ± 0.25	8.309	<0.001
FC between Cerebelum_4_5_L and ANG.R	0.24 (0.14, 0.41) ‡*	0.01 (−0.16, 0.08) †	0.14 (0.04, 0.25)	0.07 (0.01, 0.16) †	25.823	<0.001
FC between Cerebelum_4_5_R and ANG.R	0.28 (0.12, 0.37) ‡§*	0.01 (−0.12, 0.08) †	0.14 (0.01, 0.22) †	0.07 (−0.04, 0.24) †	22.317	<0.001
FC between Cerebelum_9_L and ORBmed.L	0.38 ± 0.20‡	0.56 ± 0.12†§	0.34 ± 0.18‡	0.44 ± 0.17	5.498	0.002

Note: The nomenclature for cerebellar regions follows the AAL atlas and has been retained as provided. Post hoc pairwise comparisons ($p<0.05$): * vs. controls; † vs. COMISA group; ‡ vs. insomnia group; § vs. OSA group.

Abbreviations: Oper, opercular; Mid, middle; R, right; L, left; fALFF, Fractional Amplitude of Low-Frequency Fluctuations; DC, Degree Centrality; FCD, Functional Connectivity Density; ANG, Angular Gyrus; FFG, Fusiform Gyrus; IFG, Inferior Frontal Gyrus; MFG, Middle Frontal Gyrus; MOG, Middle Occipital Gyrus; MTG, Middle Temporal Gyrus; ORBmed, Medial Orbital Gyrus; SFG, Superior Frontal Gyrus; TPO, Temporal Pole.
